# A systematic review of the role of culture in the mental health service utilisation among ethnic minority groups in the United Kingdom

**DOI:** 10.1017/gmh.2022.2

**Published:** 2022-02-22

**Authors:** Sandra Chidimma Nwokoroku, Barbara Neil, Chris Dlamini, Vivian Chinonso Osuchukwu

**Affiliations:** 1 School of Health and Wellbeing, Teesside University, Middlesbrough TS1 3BX, UK; 2 School of Nursing and Health Sciences, University of Sunderland, Sunderland SR1 3SD, UK

**Keywords:** Culture, ethnic minorities, immigrants, mental health service, service utilisation

## Abstract

Although mental health (MH) services and psychological support are tailored to fit the MH needs of those requiring these services in the UK, underutilisation persists. Current evidence suggests that ethnic minorities underutilise MH services with culture implicated in this trend. However, there is limited evidence from systematic reviews integrating the findings of primary studies on the role of culture in MH service utilisation among ethnic minorities. This review aims to synthesise and summarise evidence on the role of culture in MH service utilisation among ethnic minorities in the UK. Two reviewers searched CINAHL, APA PsycINFO and Medline databases using the Preferred Reporting Items for Systematic Review and Meta-Analysis. Two other reviewers screened the abstracts and full text, while three conducted data extraction and assessed study quality using the Critical Appraisal Skills Programme checklist for qualitative studies. One's culture was repeatedly identified to play a role in MH service utilisation among the ethnic minorities in the UK. The impact of cultural factors on service utilisation was through its effects on structure/institution, beliefs, stigma and perception of service. In addition, discrimination and other racism-related negative experiences during service use were found to inform perception and use of MH services. These findings suggest that MH services should be tailored to cultural differences to optimise service utilisation.

## Background

The UN Department of Economic and Social Affairs (UNDESA) (International Migration Report 2017) has reported a steady increase in global migration within the last two decades. As a result, populations such as the UK continue to experience increased diversity, partly explained by international migration. In addition, the literature suggests an association between migration and mental health (MH) disorders among the UK population (Carta *et al*., 2005; Winkelman *et al*., 2013).

MH disorder is a global public health burden that influences employment, morbidity and mortality (Evans-Lacko *et al*., 2013; Chesney *et al*., 2014; Saxena, 2018; Vahdaninia *et al*., 2020). Saxena (2018) reported the need for studies and interventions to identify priority groups and reduce risk factors to MH disorders to reduce the disease burden. One of the groups identified as a priority is the members of ethnic minority groups.

The UK Department of Health (2005) prioritised MH service provisions for ethnic minority communities to ensure equality in service utilisation. Race Relations (Amendment) Act of 2000 imposes responsibilities on public sector institutions ensuring racial equality and access to public services in the UK. As a result, the UK government developed the National Service Framework for Mental Health (1999) for combating any form of discrimination against those that require MH services, including ethnic minority groups. Furthermore, the Delivery Race Equality in Mental Healthcare (DRE) action plan was developed to ensure equality and remove discrimination against Black, Asian and Minority Ethnics (BAME) in the use and provision of MH service in England (Memon *et al*., 2016). As a result, MH and specialist psychological services such as Mental Health and Psychosocial Support, interventions for prevention and treatment of psychiatric disorders, have been integrated into MH services. In addition, the UK Department of Health mainstreamed other psychological therapies such as cognitive behaviour therapy (CBT) and the use of psychoactive drugs into MH care (Satinsky *et al*., 2019).

Despite these available therapies and community-based care, ethnic minority groups in the UK underutilise MH service (Broman, 2012; Furnham and Hamid, 2014; Miranda *et al*., 2015; Maciagowska and Hanley, 2018). A systematic review by Goodman *et al.* (2008) compared the population-based prevalence of child MH disorders between the white British and the ethnic minority groups. Their study reported a difference in MH disorders between the children of the white British and the ethnic minority groups and possible unmet MH service needs of the ethnic minority groups (Goodman *et al*., 2008). In addition, the study found a gap in cross-cultural reasons that might account for differences in MH service use between the whites and the ethnic minority communities. To better understand the reasons for this reported cultural variation, there is a need to explore the lived MH health service utilisation experience of people from an ethnic minority group.

Culture may affect perceptions and the uptake of MH services (Aloud and Rathur, 2009; Edge and MacKian, 2010; Shefer *et al*., 2013; Rabiee and Smith, 2014; Memon *et al*., 2016). Culture is a way of life, identity and social values. In MH utilisation, we define culture as a set of language, identity and race governed by social norms that contribute to an individual's view of MH. However, there are inconsistencies in reports on the role of culture in MH service utilisation among adults from ethnic minority groups in the UK. This gap in the literature on the impact of culture in MH service use prompted this review to integrate the existing evidence on this subject.

## Objective

Our study aimed to synthesise and summarise experiential evidence on the role of culture in MH service utilisation among ethnic minority groups in the UK with the following objectives:

- To conduct an extensive review of the literature on the role of culture in MH service utilisation among ethnic minority groups in the UK.
- To critically evaluate the quality of evidence on the role of culture in MH service use among ethnic minority groups in the UK.

The review question was, does culture play a role in the MH services utilisation among ethnic minority groups in the United Kingdom?. This research team adopted the patient/population, intervention, comparison and outcomes (PICO) framework to develop this review question, as shown in Table 1.

**Table 1.** PICO framework

PICO	Inclusion	Justification	Exclusion
Population	Adult in the UK. Aged 18+. Member of an ethnic minority	MH problems are likely developed in adulthood (Kessler *et al*., 2005). Members of the ethnic minority group have been reported to underutilise MH service (Broman, 2012; Furnham and Hamid, 2014; Miranda *et al*., 2015; Maciagowska and Hanley, 2018)	Studies conducted outside the UK. Studies that were conducted in the UK but not with ethnic minorities. Studies conducted with children
Interest	Culture	Culture is the variable of interest in this review	Studies that measure other variables in relation to culture and not solely culture
Outcome	MH service utilisation	This is to ensure that only their MH service use is measured	Studies that measure other MH factors other than its utilisation were excluded
Additional criteria	Studies conducted between 2010 and 2020	The evidence on migration showed that the UK experienced an increase in the migrant population by 1.4 million which is about 5.5% of the national population in the last decade (Hawkins, 2016)	Secondary studies on MH utilisation of immigrants such as a systematic review. Reported views of others such as health professionals Studies that only their abstracts were available. Studies that do not meet the quality and risk of bias screening

## New contributions

No review exists on the role of culture in MH service utilisation among the ethnic minority groups in the UK. Due to this gap, our systematic review will be of value in policy, practice and MH intervention development. Our findings add to solid evidence and enhance the understanding of the role of culture in MH service utilisation among the ethnic minority groups in the UK.

## Method

### Guidelines and study registration

The Preferred Reporting Items for Systematic Reviews and Meta-Analyses (PRISMA) guidelines was adhered to in this review. In addition, we registered the protocol for this study on the International Prospective Register of Systematic Reviews (PROSPERO) (registration number: CRD42020206615).

### Search strategy

A vital part of this search strategy was using index Medical Subject Headings (MeSH) terms (as shown in Table 2). In addition, we searched CINAHL, APA PsycINFO and Medline. Two researchers carried out an extensive search of the selected databases with support from a health science librarian to identify the literature relevant to our research question (Cooper *et al*., 2018). Furthermore, a hand search of grey literature (journals on migration) and screening the reference list of all included studies were carried out (Bettany-Saltikov, 2010). The Boolean logic ensures this review's reliability, specificity and replicability. Finally, we included search terms related to ethnic minority groups AND the UK AND adults aged 18+ AND culture AND Mental health service utilisation as shown in Table 2.

**Table 2.** Search strategy

PICO	Boolean operators	
	AND	OR
Population	Aged 18+. Member of an ethnic minority	Immigrants, Refugee, Asylum seeker, Ethnic minorit*, Migrants, Minorit* groups, Ethnic group, Racial minorit*, emigrants, Immigration
	The UK	United Kingdom, England, Scotland, Wales, Northern Ireland, Britain
Interest	Culture	Cultur*, Social behaviour, Social behavior, social habits, crosscultur*, cross culture*, customs, traditions, language, religion, social characteristics, social norms, morals, beliefs, ‘way of life’
Outcome	Mental health service utilisation	Mental health, Mental service, Counselling, CBT, Cognitiv* behav* therap*, Psycholog* therap*, Samaritans, Psychiatr*, IAPT, Psychotherap*

This table shows PICO questions and their key search words.

### Information sources

Tentatively eligible studies were obtained by searching CINAHL, APA PsycINFO and Medline. The databases and reference list search were started in May and concluded by the 31st of July 2020.

### Study selection

The population included in this review is adults (18+ years), ethnic minority population in the UK. There is evidence that MH illnesses are developed in adulthood, which informed adulthood criterion (Kessler *et al*., 2005). This review included studies published from 2010. The evidence on migration shows that the UK experienced an increase in migrant population by 1.4 million (5.5% of the national population) in the last decade justified this criterion (Hawkins, 2016).

Most of the available evidence on MH service utilisation among ethnic minorities is qualitative studies and experiential (Priebe *et al*., 2016). Therefore, qualitative studies were selected in this review to explore and synthesise the evidence on lived experience from an individual perspective.

The inclusion criteria in this study are studies that measured the impact of culture, MH service, reported on qualitative methodology, published between January 2010 and July 2020 in the UK and written in the English language. In addition, we excluded articles that were (a) conducted on MH utilisation but not directly on the ethnic minority in the UK; (b) secondary studies on MH utilisation of immigrants such as a systematic review (c) reported views of others such as health professionals or (d) that only their abstracts were available. Two researchers independently conducted the title and abstract sifting. Three researchers independently assessed full text for eligibility.

### Data extraction

Data extraction was conducted independently by three reviewers. Data were extracted from the eligible studies using a predeveloped data extraction table by Caldwell *et al*. (2010). The data extracted were information on the author(s), titles, year of publications, objectives, participants, sample size, settings, methods and the relevant study findings as shown in Table 3 (Caldwell *et al*., 2010). The three researchers discussed any discrepancy and reached a consensus. A fourth researcher ran through consensus decisions.

**Table 3.** Data extraction table

Authors, date and country and focus of study	Study design	Participants, recruitment and sampling methodology	Intervention/focus of study	Findings	Key themes	Critique
Edge and MacKian (2010) Ethnicity and mental health encounters in primary care: help-seeking and help-giving for perinatal depression among Black Caribbean women in the UK	Qualitative description using an in-depth interview	12 Black Caribbean women were purposively sampled from a larger study. They were recruited from a community clinic and a large teaching hospital in the North of England	Exploring the approach of Black Caribbean women to help-seeking and their experience of seeking professional help	Black Caribbean women's configuration or no configuration of depressive symptoms is a reflection of how the help-givers perceive them	Approaches to help-seeking and structural (e.g. resistance to psychiatric labelling) and professional barriers to receiving help	The research objective was not stated The suitable study design was adopted The study was ethically approved Informed consent was obtained Participant's identity anonymised Overall: good
Shefer *et al.* (2013) Our community is the worst’: The influence of cultural beliefs on stigma, relationships with family and help-seeking in three ethnic communities in London	An exploratory qualitative study using focus group discussions	10 focus groups comprising London-based African descents. With five service users and five laypersons from the BME community per group. Participants were recruited from three dominant groups of South Asia, Black Africa and the Black Caribbean. They were recruited from community organisations based in London. Focus group discussion was done twice on separate days	Investigate cultural belief attitude and behaviour towards people of BME with mental illnesses. To explore the response of BME groups to time of change campaign	Cultural belief influences help-seeking among BMEs	Self-critical voice (internal stigma), medical critical voice (external stigma), relationship with self	Research aim clearly stated Minority ethnic groups represented Ethical approval not reported Limitation: the language barrier Overall: good
Memon *et al.* (2016) Perceived barriers to accessing mental health services among black and minority ethnic (BME) communities: a qualitative study in Southeast England	Qualitative method using an interview	26 adults from the BME community were identified from the BME community partnership centre. They were grouped into 2 focus groups	Determine barriers to accessing MH services by people from BME communities	The study identified religion as the most prominent sociocultural factor that shapes attitudes towards breast cancer and its screening. Other identified factors include family and traditional belief	Personal factors and environmental and financial factors. Factors that affect the relationship between service users and providers	Research objective clearly stated Appropriate study design Ethical approval not reported Overall: good study
Rabiee and Smith (2014) Understanding mental health and experience of accessing services among African and African Caribbean Service users and carers in Birmingham, UK	A qualitative design involving in-depth interview	49 carers and service users of Black African and African Caribbean communities were purposively sampled from established groups. 9 focus groups and four individual interviews	Understanding MH and the extent services meet the needs of the black African and African Caribbean communities	Mental illness was associated with racism and inappropriate treatment to mental illness for BMEs	Lack of respect to religious, health and spiritual beliefs, engaging users and carers in the care pathway, the importance of spirituality in healing	Aim of the study written Sampling strategy reported Appropriate study design adopted Overall: good study
Mantovani *et al*. (2017) Exploring the relationship between stigma and help-seeking for mental illness in African-descended faith communities in the UK	A qualitative study using semi-structured interviews	26 adult faith-based groups in South London using purposive convenient sampling	Ways in which stigma influences help-seeking for mental illness among African-descended communities	Adults that require MH service might experience triple jeopardy (in terms of stigma)	Belief about the cause of mental illness, silencing of mental illness due to stigma and stigma at the community level	Well defined study objective Appropriate study design Enough sample size Obtained Participants consent got Overall: good

This table shows the data extraction sheet showing all information extracted from the studies included in this review as discussed in Section ‘Risk of bias’.

### Risk of bias

The studies included in this review were of qualitative design, so the Critical Appraisal Skill Programme (CASP) (Singh, 2013), a checklist for qualitative studies, was used to assess the studies, as shown in Table 4. The CASP is a 10-item quality assessment checklist that systematically helps the researcher think about the issues captured in qualitative research. Two reviewers screened the articles using the CASP checklist guideline with the first two questions: ‘Was there a clear statement of the aims of the research?’ and ‘Is a qualitative methodology appropriate?’. Studies that answered ‘yes’ to both questions were adopted for a full appraisal and studies which answered ‘no’ to both questions were dropped (CASP, 2017). The researchers assessed the five studies included in this systematic review for trustworthiness using these four frameworks: credibility, dependability, transferability and confirmability (Guba, 1981; Lincoln, 2001; Shenton, 2004; Silverman, 2015; Trochim *et al*., 2016). As a result, all included studies report trustworthiness.

**Table 4.** Result of quality score

Authors	Durà-Vilà and Hodes (2012)	Shefer *et al*. (2013)	Memon *et al*. (2016)	Edge and MacKian (2010)	Rabiee and Smith (2014)	Mantovani *et al*. (2017)
Was there a clear statement of the aims of the research?	No	Yes	Yes	No	Yes	Yes
Is a qualitative methodology appropriate?	No	Yes	Yes	Yes	Yes	Yes
Was the research design appropriate to address the aims of the research?	Yes	Yes	Yes	Yes	Yes	Yes
Was the recruitment strategy appropriate to the aims of the research?	Yes	Yes	Yes	Yes	Yes	Yes
Was the data collected in a way that addressed the research issue?	Yes	Yes	Yes	Yes	Yes	Yes
Has the relationship between the researcher and participants been adequately considered?	Not included	Not included	Not included	Not included	Not included	Yes
Have ethical issues been taken into consideration?	Yes	Yes	Not included	Yes	Yes	Yes
Was the data analysis sufficiently rigorous?	No	Yes	Yes	Yes	Yes	Yes
Is there a clear statement of findings?	Yes	Yes	Yes	Yes	Yes	Yes
Is the research valuable?	Yes	Yes	Yes	Yes	Yes	Yes

This table shows the result for the quality score for selected qualitative articles using the Critical Appraisal Skills Programme (CASP, 2017) as explained in Section ‘Data extraction’.

After quality assessment, the reviewers excluded one study as it did not meet up to 70% on the checklist (Treloar *et al*., 2000). A total of five studies were of high quality and included in the systematic review (Edge and MacKian, 2010; Shefer *et al*., 2013; Rabiee and Smith, 2014; Memon *et al*., 2016; Mantovani *et al*., 2017).

### Data synthesis and analysis

The researchers adopted thematic analysis in synthesising the findings from the five included studies. The synthesis of results of the included studies in this review shows cultural factors such as structure (three studies), perception and beliefs of service users (four studies), cultural barrier (four studies) and stigma (five studies). In this synthesis process, a description of studies, tabulation and thematic analysis was carried out in stage five and reported in the sixth stage.

### Characteristics of included studies

**Fig. 1.**
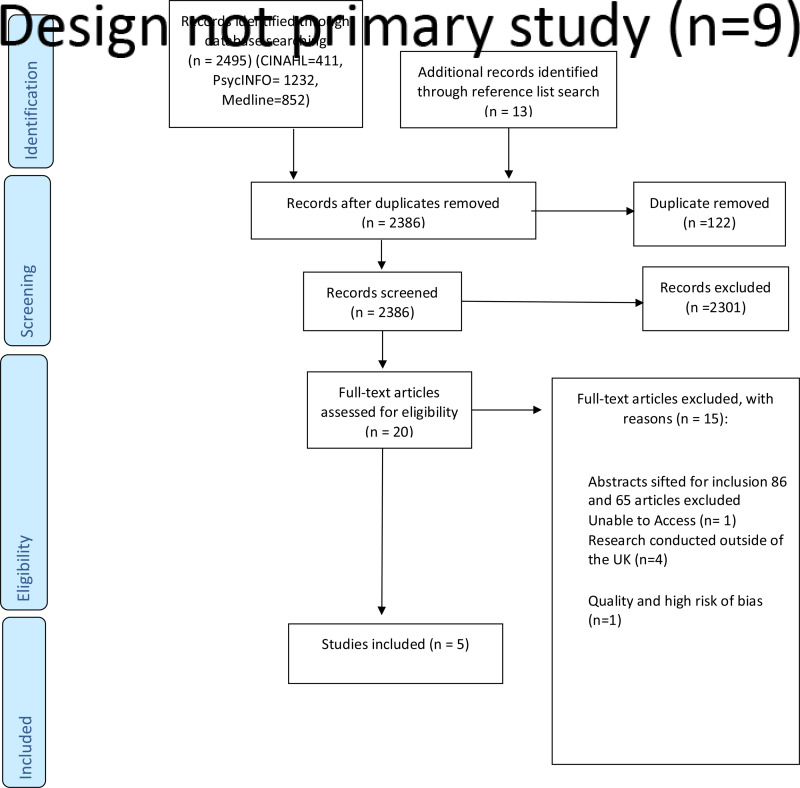
PRISMA flow chart.

The search from four databases yielded 2508 references, as presented in a PRISMA flow chart in Fig. 1. The removal of duplicates followed this. Next, the title and abstract were sifted, followed by the assessment of 20 full papers. After quality assessment, we excluded one study. Finally, the full text of the five studies was printed and studied. The summary of the included studies is given in Table 5.

**Table 5.** Summary of included studies

Study	Aim	Setting	Participants		Data collection	Data analysis
			Sample size	Ethnicity		
Shefer *et al.* (2013)	To extend our knowledge about cultural beliefs surrounding mental illness held by the predominant BME communities in London; and to analyse how these beliefs influence experiences of stigma and relationships with family for individuals with mental illness in these communities	BME communities in London	10	Three predominant ethnic minority groups in London	A qualitative approach, using focus group	Thematic and content analysis
Memon *et al.* (2016)	To understand the perception and to inform culturally effective MH service to BME	Southeast England	26	BME community members	Qualitative method via focus group discussion	Thematic analysis
Edge and MacKian (2010)	To examine the prevalence and psychosocial risks for perinatal depression among this ethnic group	London	12	Black Caribbean women	A qualitative study using in-depth interviews	Thematic analysis
Rabiee and Smith (2014)	To explore the extent to which MH service provision meets the need of African and African Caribbean in the UK	Brighton	49	African and African Caribbean	A qualitative study via interviews	Transcripts were analysed using Krueger's framework and Rabiee's guidelines (Krueger and Casey, 2000 Rabiee and Smith, 2014)
Mantovani *et al*. (2017)	Ways in which stigma influences help-seeking for mental illness among African-descended communities	London	26	Adults faith-based groups in South London using purposive convenient sampling	A qualitative study using semi-structured interviews	

This table shows the summary of all studies included in this review as explained in Section ‘Data extraction’.

## Results

The themes reported to affect MH service utilisation in this review are the following.

### Structural factors

The structural factor theme involves factors found in the society that influence the MH utilisation of the patient. Factors found were nature and awareness of MH service, language, cultural competence of service, waiting times in service delivery, the attitude of staff, professional stigma against BAME and social stress (Edge and MacKian, 2010; Rabiee and Smith, 2014; Memon *et al*., 2016). The majority of the included studies made these findings. This synthesis found that structure accounts for the perception of service delivery and willingness to use the available MH services. Rabiee and Smith (2014) reported a lack of respect for their culture, belief and religion by the MH service delivery. These three areas of the life of the patient have been suggested by the Department for Health UK to play a critical therapeutic role in MH service among people of an ethnic minority but ignored according to this review evidence (Leavey *et al*., 2007; Poole and Cook, 2011; Rabiee and Smith, 2014). For instance, one of the participants reported that MH services should be ‘Seeking to understand people, their personal circumstances, their culture, and their beliefs – not simply dishing out medication’ (Rabiee and Smith, 2014). This finding supports evidence that structure can be a constraint in help-seeking, especially if the cultural disparity is present (Munro *et al*., 2007).

### Perception and belief of service users

The perception of MH service by service users theme had subthemes of the relationship between the service provider and the user, service user attitude and behaviour, personal experiences of using MH service, self-critical voice and difficulty in conceptualising mental illness (Edge and MacKian, 2010; Shefer *et al*., 2013; Rabiee and Smith, 2014; Memon *et al*., 2016). Four out of the five included studies reported that the patient's perception affects MH service utilisation. Issues such as families hiding a member living with a mental illness due to shame to protecting family reputation from gossip may account for the perception, meaning of mental illness in their culture and attitude towards seeking mental help (Shefer *et al*., 2013). This finding contributes to the literature that cultural definition of shared value and idea towards mental illness also affects the perception and attitude of members of that culture to MH. Both positive and negative experiences of using MH services were reported by Rabiee and Smith (2014). The feeling of inadequacy and guilt accompanied MH service use, which was said to be aggravated by the nature of the operation of MH service. For example, patients described sectioning the MH unit as ‘criminalising black people’ (Rabiee and Smith, 2014). Edge and MacKian (2010) also reported that patients who have had a negative encounter with MH service use are reluctant to return. Service users’ perception is closely linked to their beliefs, as reported in this study. The patient's belief is a theme reported by all included studies in this review to influence MH service utilisation. Subthemes here were belief about the cause of illness, diagnosis of illness, silencing mental illness, sociocultural belief about mental, cultural belief about mental illness and recognition of MH problems (Edge and MacKian, 2010; Shefer *et al*., 2013; Rabiee and Smith, 2014; Memon *et al*., 2016; Mantovani *et al*., 2017). Rabiee and Smith (2014) found the theme of lack of respect for spiritual and religious beliefs to cut across their participants. Shefer *et al*. (2013) reported that cultural belief affected not just MH utilisation but relationships of the BAME. These findings support previous evidence that belief about MH service affects its effectiveness and influences service utilisation (Dupree *et al*., 2010; Guzman *et al*., 2015). Another review suggested that personal interpretation and belief about the causes of mental illness and diagnosis can act as a filter for healthcare service information given and affect decisions (Munro *et al*., 2007). These linked themes across the studies support the literature that a patient's understanding and belief about a treatment given influences adherence to the treatment (Munro *et al*., 2007).

### Stigma due to cultural differences

The last theme in this review was stigma, as reported by all five studies included in the synthesis. Resistance to psychiatric labelling, cultural identity and stigma, negative experience and racism, production of stigma and internal and external stigma were subthemes reported under stigma (Edge and MacKian, 2010; Shefer *et al*., 2013; Rabiee and Smith, 2014; Memon *et al*., 2016; Mantovani *et al*., 2017). In their study, Mantovani *et al*. (2017) defined stigmatising as a form of discrimination that occurs in the circumstance of power as seen in a service provider and user relationship. They found that stigma affects how MH service is perceived and utilised. The participants in the study by Rabiee and Smith (2014) expressed concern about racism and the stigma that they experienced from service providers when they went to seek help. For example, an African reported that a GP asked, ‘Why are you all Somalians in the UK?’ (Rabiee and Smith, 2014). The participants felt stigmatised and described this as a reason for not utilising MH services. Shefer *et al*. (2013) also reported stigma and stigmatising attitudes across the ethnic minority communities in their study. Edge and MacKian, (2010) found psychiatric labelling and how it can reduce the uptake of MH services by people of the ethnic minority communities in the UK. This finding is consistent with previous studies that found discrimination and stigma about MH illness across ethnic groups, especially for mixed and black groups (Gabbidon *et al*., 2014). In another study on stigma, a high level of stigma was found against mental illness in all cultures, even though its nature and consequences differ across cultures (Koschorke *et al*., 2017).

### Overall cultural barriers

A cultural barrier was a theme that four of our five studies reported. It had subthemes of cultural competence, staff attitude, cultural naivety, negative experiences, cultural belief, cultural insensitivity, cultural perception of family shame due to illness and discrimination (Edge and MacKian, 2010; Shefer *et al*., 2013; Rabiee and Smith, 2014; Memon *et al*., 2016). This synthesis found that these linked subthemes affect the decision of the ethnic minority communities to seek professional mental help. This finding focuses on the type of service people of diverse cultures are presented with within the MH system. When interventions are not designed to consider cultural diversity, some might contradict people's culture. These patients in these studies reported that they would not go back to a clinic where the practice contradicts their culture. This review finding supports the study by O'Mahony *et al*. (2012) that the cultural background, cultural differences and social stigma of the ethnic minority influence MH service seeking behaviour.

## Discussion

### Review of the findings

This study aims to comprehensively explore and synthesise evidence to answer the question of the role of culture in MH service utilisation among people of ethnic minority groups in the UK. We included five studies in this review, with 166 participants. The studies identified and included in this study were all qualitative. There is an increase in the volume of qualitative studies conducted in clinical and health care research and the need to synthesise the themes identified in the literature (Munro *et al*., 2007; Cohen and Crabtree, 2008). Our review found that culture plays a vital role in MH service utilisation among the ethnic minority groups in the UK. A possible explanation is a report from BAME patients that MH services were designed without considering the cultural norms and values of the ethnic minority groups (Rabiee and Smith, 2014). The present nature of the MH services may account for the reported low MH service utilisation by the BAME groups. MH service providers should tailor services to understand patients more by considering their circumstances and culture. These findings are consistent with another systematic review from the US population by Derr (2016). However, our findings expand on the existing evidence by revealing four aspects of culture that affects MH service utilisation by the BAME groups in the UK.

One of the cultural factors found to play a role in MH service utilisation is the structure of MH services. Structural factors reported in this review are the nature of the service, the design and mode of service delivery and how compliant the interventions are with the cultural values of the BAME. This finding is consistent with the model on MH by Corrigan *et al*. (2014) that structural factors may act as systemic barriers to MH service use.

The majority of the studies we reviewed in this study shows that the perception of both the patients, family community members about MH affected help-seeking. Our finding is consistent with the literature that attitude and perception about MH may influence MH service use (Gaston *et al*., 2016). In addition, factors such as experience in service use and cultural interpretation of MH accounted for the formation of these perceptions. Closely linked to this is the finding that the belief of the ethnic minority group might affect their MH service use (Jimenez *et al*., 2012). Specifically, the faith and the explanatory model on the cause of MH illness vary across cultures (Jimenez *et al*., 2013). The findings of this study expanded on the evidence by identifying the aspects of the belief, such as belief about the cause of illness, diagnosis, silencing and recognition of mental illness that might affect MH use by ethnic minority groups in the UK. Therefore, MH services should be extended to patients' families to create more insight, understanding and acceptance of MH illness among the BAME group.

Even though self-stigma is a stronger predictor of MH service use than public stigma, this review found public and self-stigma mitigating against MH service use among ethnic minority groups (Nam *et al*., 2013; Wu *et al*., 2017). This finding is consistent with previous studies that found discrimination and stigma about MH illness across racially ethnic groups, especially for mixed and black groups (Gabbidon *et al*., 2014). In another study on stigma, a high level of stigma was found against mental illness in all cultures, even though its nature and consequences differ across cultures (Koschorke *et al*., 2017). The participants in our included studies felt stigmatised, as shown in some of the comments in the result section. Systemic issues such as psychiatric labelling and reported racism may discourage the BAME community from seeking help in mental illness cases. The MH service should be tailored and delivered to avoid stigma to the end-users of patients.

### Knowledge gap

This review outcome shows that cultural factors affect MH service utilisation among ethnic minority groups. The evidence suggests that subthemes of cultural competence, staff attitude, cultural naivety, negative experiences, cultural belief, cultural insensitivity, cultural perception of family shame due to illness and discrimination affect MH service utilisation. Although this is experiential evidence, there is a need for a quantitative examination of this topic to understand if this is transferable to a larger population. There is also a need for this study to be replicated among younger adults. Finally, there is a need to investigate if there is an impact of COVID-19 on MH service utilisation among this sample.

### Strength and limitation of the review

The primary strength of the approach adopted in this systematic review is using qualitative methodology articles. The qualitative study allows for an in-depth understanding of the experiences of ethnic minority groups on MH service utilisation. Alongside this strength also comes the limitations of this study. First, all studies included in this synthesis were from the UK population; the findings can only apply to the MH service of ethnic minority groups in the UK. Also, studies included in this review were those conducted from 2010 to 2020, which can be a limitation. This review targeted studies that recruited people of an ethnic minority across the UK. Still, the studies included were conducted in London and Birmingham to represent the entire UK population. There were limited studies on MH service by the ethnic minority communities in other parts of the UK except for London and Birmingham.

## Conclusion

This study will be the first systematic review of literature evidence on the role of culture in MH service utilisation among ethnic minority groups in the UK. This systematic qualitative review shows that culture plays a vital role in utilising MH services among people of ethnic minority communities. Cultural factors such as professional and structural barriers, perception of MH service, beliefs, cultural barriers and stigma could be inhibiting BAME who require MH service from using the service. The second objectives of this review were to evaluate the quality of the existing evidence and synthesise and summarise the role of culture in MH service use. This review shows the need to prioritise cultural consideration in developing and implementing MH interventions. One therapeutic model cannot serve all in a culturally diverse society such as the UK. Ethnic community members should be key actors in formulating MH interventions to ensure cultural compatibility and enhance utilisation.
